# Regulation of T-cell Tolerance by Lymphatic Endothelial Cells

**DOI:** 10.4172/2155-9899.1000242

**Published:** 2014-07-31

**Authors:** Sherin J. Rouhani, Jacob D. Eccles, Eric F. Tewalt, Victor H. Engelhard

**Affiliations:** Carter Immunology Center and Department of Microbiology, Immunology, and Cancer Biology, University of Virginia School of Medicine, Charlottesville, VA 22908, USA

**Keywords:** Lymphatic endothelial cells, Peripheral tissue antigens, T-cell tolerance, Inflammation, Lymph node stromal cells, PD-L1

## Abstract

Lymphatic endothelial cells are most often thought of as structural cells that form the lymphatic vasculature, which transports fluid out of peripheral tissues and transports antigens and antigen presenting cells to lymph nodes. Recently, it has been shown that lymphatic endothelial cells also dynamically respond to and influence the immune response in several ways. Here, we describe how lymphatic endothelial cells induce peripheral T-cell tolerance and how this relates to tolerance induced by other types of antigen presenting cells. Furthermore, the ability of lymphatic endothelial cells to alter immune responses under steady-state or inflammatory conditions is explored, and the therapeutic potential of bypassing lymphatic endothelial cell-induced tolerance to enhance cancer immunotherapy is discussed.

## Antigen Presenting Cells and T-cell Tolerance Induction in the Thymus and Lymph Nodes

T-cell receptors (TCR) are stochastically generated in the thymus, which enables the immune system to recognize a tremendous diversity of foreign antigens. However, it also creates T-cells with the potential to recognize and attack host tissues expressing normal self-antigens. To prevent autoimmunity, T-cells that recognize self-antigens are tolerized through intrinsic mechanisms such as deletion, anergy, and induction of a regulatory T-cell (T_reg_) phenotype, or through extrinsic suppression by already differentiated T_reg_ [1]. Tolerance induction occurs in the thymus at the time of T-cell development, but also occurs in lymph nodes (LN) draining peripheral tissues. Deletion and T_reg_ induction are thought to be the dominant mechanisms of tolerance in the thymus, while anergy, deletion and T_reg_ induction all occur in LN [2]. In all cases, tolerance induction is driven by antigen presenting cells (APC) that present ubiquitously expressed and tissue-restricted antigens. While it was initially thought that dendritic cells (DC) were the primary tolerance-inducing APC, it has become clear recently that several other cell types can also serve this role. Among them are the lymphatic endothelial cells (LEC) that reside in LN.

## Direct Expression of Peripheral Tissue Antigens by LEC and Other APC

All APC have the ability to present antigens derived from ubiquitously expressed proteins, but vary in their ability to present peripheral tissue antigens (PTA), which are defined as antigens from proteins normally expressed in fewer than 5 tissues [3]. A particular challenge has been to understand how tolerance to PTA would develop, since it is expected that these antigens will be largely absent from the thymus. DC are particularly efficient at acquiring and presenting antigens, including PTA, that are originally derived from other cells, both in the thymus [4] and LN [5–7]. This led to early models suggesting that tolerance to PTA occurred primarily in LN, and was based on DC acquisition of PTA derived from the tissues drained by individual LN [8]. However, it was subsequently shown that medullary thymic epithelial cells (mTEC) transcriptionally express PTA in the thymus [9,10]. More recently, several groups including our own, have shown that multiple subpopulations in LN also transcriptionally express PTA. PTA are expressed by extrathymic Aire expressing cells (eTAC) and several LN stromal cell (LNSC) subsets including LEC, blood endothelial cells (BEC), and fibroblastic reticular cells (FRC) [11–13]. Interestingly, each of these subpopulations presents distinct PTA, although the overall size and overlap of their PTA repertoires has not been determined. Nonetheless, this mechanism broadens the presentation of PTA to non-draining LN, and enables efficient system-wide peripheral tolerance induction.

The transcriptional regulation of PTA expression is best understood in mTEC in the thymus, where it is controlled by the autoimmune regulatory element (Aire) [10]. Using single cell PCR assays, it was demonstrated that Aire stochastically induces expression of a PTA in 1–3% of total mTEC [14,15]. Limiting the number of PTA expressed in each cell is likely advantageous to ensure that each antigen is adequately represented on the limited number of MHC molecules on the cell surface, and avoid possible deleterious consequences for mTEC functionality from expressing a large number of functionally specialized proteins. Some PTA transcripts have different start sites in mTEC compared to peripheral tissues, and Aire-regulated genes within a cell are clustered based on chromosomal position rather than by cell of origin [3,14,16]. These results suggest than Aire operates by opening up a region of the chromosome to additional transcriptional regulators, rather than inducing mTEC to differentiate towards and express PTA from an alternative cell lineage. Interestingly, while Aire also regulates PTA expression in eTAC, the PTA repertoires of mTEC and eTAC are distinct [11], suggesting that these additional transcriptional regulators may differ between the two cell types.

The transcriptional regulation of PTA in LNSC is not well understood. Aire is not expressed in LEC, FRC, or BEC [12,13], and expression of PTA in these subsets does not change in Aire^−/−^ mice [12]. Aire is a member of the SAND family of transcription factors [17], and Yip et al. [18] demonstrated that Deaf1, another SAND family member, controls the expression of 600 genes in LNSC of pancreatic LN. Most of these genes were distinct from genes regulated by Aire in eTAC. Deaf-1 regulated genes included pancreatic polypeptide (Ppy) and insulin 2 (Ins2), which are PTA normally expressed in pancreatic islets. Ppy and Ins2 are primarily expressed in LEC and FRC, respectively, and at much lower levels in CD31^neg^podoplanin^neg^ cells [12,19]. However, Deaf1 is expressed in all LNSC subsets [13] suggesting that additional transcriptional regulators that differ among these subpopulations control the specificity of PTA expression. Deaf1 also controls PTA translation by regulating the transcription of the eukaryotic translation initiation factor *Eif4g3* [19]. Further work is needed to determine the extent to which Deaf1 controls expression of PTA in non-pancreatic LN, and whether other members of the SAND family also play a role in controlling PTA expression in LEC and other LNSC. The overall PTA repertoire of LEC and other LNSC remains to be determined, as does the pattern of PTA expression in individual LECs. While PTA expression in mTEC provides a logical model for how PTA expression in LEC may operate, the master transcriptional regulator is different and future studies will illuminate what other similarities and differences exist.

## Cross-presentation of Soluble and Tumor Derived Antigens Acquired by LEC

In addition to transcriptionally expressed PTA, LEC can also acquire and cross-present exogenously derived antigens. Lund et al. [20] demonstrated that LEC in tumor-draining LN can acquire antigens derived from VEGF-C overexpressing tumors, and present them via their MHC I molecules. Further work from the same group demonstrated that LEC engulf intradermally injected OVA, and present OVA antigen to T-cells *in vitro* [21]. Previous work has shown that soluble antigens travel to the LN through the lymphatics. While large antigens are taken up by macrophages in the subcapsular and medullary sinuses, antigens smaller than 70 kDa travel through FRC-lined conduits into the paracortex and B cell follicles where they are engulfed by DC and B cells [22–24]. LEC were found to engulf intradermally injected OVA as efficiently as DC [21], although LEC comprise only 0.5% of the total OVA^+^ cells in LN. The presentation of both tumor-derived and soluble antigens by LEC led to dysfunctional T-cell activation and increased apoptosis *in vitro* [20,21]. However, the *in vivo* consequences were not examined. In addition, while the induction of dysfunctional T-cells in the context of tumor outgrowth is intriguing and may illuminate another aspect of the immunosuppressive microenvironment in and around tumors, the results with OVA suggest that LEC might also limit responses to foreign antigens. Further work is needed to fully understand the impact of cross-presentation of acquired antigen by LEC on both immunity and tolerance.

## Mechanisms of T-cell Tolerance Induction by LEC

The demonstration that LEC express PTA and function as tolerance-inducing APC in LN was the culmination of our work over several years to understand tolerance to PTA expressed in both melanocytes and melanomas, termed melanocyte differentiation antigens, which are targets in both autoimmune vitiligo and melanoma immunotherapy. Using a model system based on recognition of one such melanocyte differentiation antigen, tyrosinase, we showed that tyrosinase-specific CD8 T-cells do not undergo central tolerance in the thymus [25]. In addition, under steady state conditions, DC in LN do not present tyrosinase. Instead, tolerance to tyrosinase is strictly due to direct expression of tyrosinase mRNA and display of tyrosinase antigen by MHC I molecules on LEC [12]. This antigen presentation leads to activation and initial proliferation of tyrosinase-specific CD8 T-cells, but these cells undergo apoptosis and deletion rather than accumulating [12,25,26].

While this process of abortive proliferation has been shown in many models of CD8 deletional tolerance, the mechanisms involved in driving this outcome have been somewhat unclear. Some previous work had established that peripheral tolerance could be induced by antigen engagement in the absence of costimulation [27–31], while other studies pointed to the engagement of inhibitory molecules [32–36]. While investigating the mechanisms involved in LEC-induced deletion, we found that both of these processes were involved and interdependent [26]. LEC do not express any of the costimulatory molecules that normally drive immunogenic accumulation of activated T-cells, such as CD80, CD86, OX40L, 4-1BBL, or CD70. However, they express multiple ligands that can activate inhibitory pathways, and express a particularly high level of PD-L1. Indeed, deletion of tyrosinase-specific CD8 T-cells is strictly dependent on engagement of the PD-1/PD-L1 pathway [26]. However, it is antigen activation in the absence of costimulation that leads to rapid, high-level upregulation of PD-1 on tyrosinase-specific CD8 T-cells, which is required for deletion to occur. This can be overcome by administration of exogenous anti-4-1BB costimulation. Importantly, tyrosinase-specific CD8 T-cells that are rescued from LEC-mediated deletion gain effector function and induce autoimmune vitiligo. Given that LEC express multiple PTA, these results suggest that impairment of LEC-induced tolerance could have a role in the induction of numerous autoimmune disorders. While there is considerable interest in PD-1/PD-L1 as a mechanism to suppress pathology in peripheral tissue and in the genesis of clonal exhaustion in tumors [37–39], these results establish a central role of this pathway in peripheral tolerance induction.

LEC that have acquired soluble OVA cross-present it to CD8 T cells in a TAP1-dependent manner, and *in vitro*, this leads to decreased OT-I IFNγ and IL-2 production and increased expression of Annexin V, PD-1, CD80, CTLA-4 [21]. Cross-presentation of tumor antigens by LEC from the tumor draining LN also leads to increased Annexin V staining on co-cultured tumor-specific CD8 T-cells *ex vivo* [20]. This demonstrates that cross-presentation of soluble antigens by LEC *in vitro* leads to dysfunctional T-cell activation, similar to the phenotype seen when LEC directly present the PTA tyrosinase *in vivo* [26]. This suggests that under steady-state conditions, the constellation of inhibitory molecules expressed by LEC combined with their lack of costimulatory molecules predisposes LEC to induce dysfunctional T-cell activation, regardless of the source of the antigen. This raises important questions about whether the tolerance-inducing state of LEC can be modulated, such as during infection when the LEC may acquire foreign antigens draining through the lymphatics.

T-cell tolerance can take many forms, including deletion, anergy, or T_reg_ induction. The form of tolerance induced has been shown to depend on several factors, including TCR avidity, availability of costimulatory or inhibitory pathways, and the cytokine environment [40–45]. mTEC, DC, and eTAC have all been shown to induce multiple forms of tolerance [2,11,46,47], while to date LEC have only been shown to induce deletion *in vivo* [26]. A question of some interest is whether LEC can also induce other forms of tolerance, based on the molecules they express or the particular microenvironmental niche in the LN that they occupy.

## LEC Interactions with DC Leading to CD4 T-cell Tolerance Induction

LEC express intermediate levels of MHC II molecules, suggesting they might also play a role in CD4 T-cell tolerance. MHC II expression is under the control of the class II transactivator (CIITA). In mice, CIITA is regulated by three different promoters (pI, pIII, and pIV) which are differentially expressed in different cell types [48]. It was recently demonstrated that LEC only express pIV mRNA [49], suggesting that endogenous expression of MHC II on LEC is controlled by CIITA driven off of this promoter. Surprisingly, LEC from pIV^−/−^ mice still express approximately half the level of MHC II as LEC from wild-type mice, suggesting that some of the MHC II on the cell surface is not endogenously synthesized by LEC. Instead, these investigators found that LEC acquire MHC II from DC *in vitro* and *in vivo*. This is highly selective, as MHC II is not acquired from macrophages or B cells, and LEC do not acquire costimulatory molecules from DC. The mechanism of transfer was found to involve cell contact and/or exosomes. Interestingly, CD4 T-cells specific for antigens displayed by the MHC II molecules on these LEC did not proliferate *in vitro*, but showed an increase in Annexin V staining, suggesting an unusual form of deletional tolerance. Furthermore, when these T-cells were subsequently restimulated with αCD3/αCD28 for an additional 3 days, they showed a reduced proliferative response. However, it is not clear whether the decrease in proliferation is due to the increased level of T-cell death observed in the first 3 days of co-culture, or if LEC additionally suppress T-cell proliferation through an antigen-dependent mechanism. The induction of tolerance induced by peptide:MHC II complexes acquired by LEC remains to be established *in vivo*, as does the importance of this modality relative to presentation of these same complexes by DC in the same LN.

**Figure 1:**
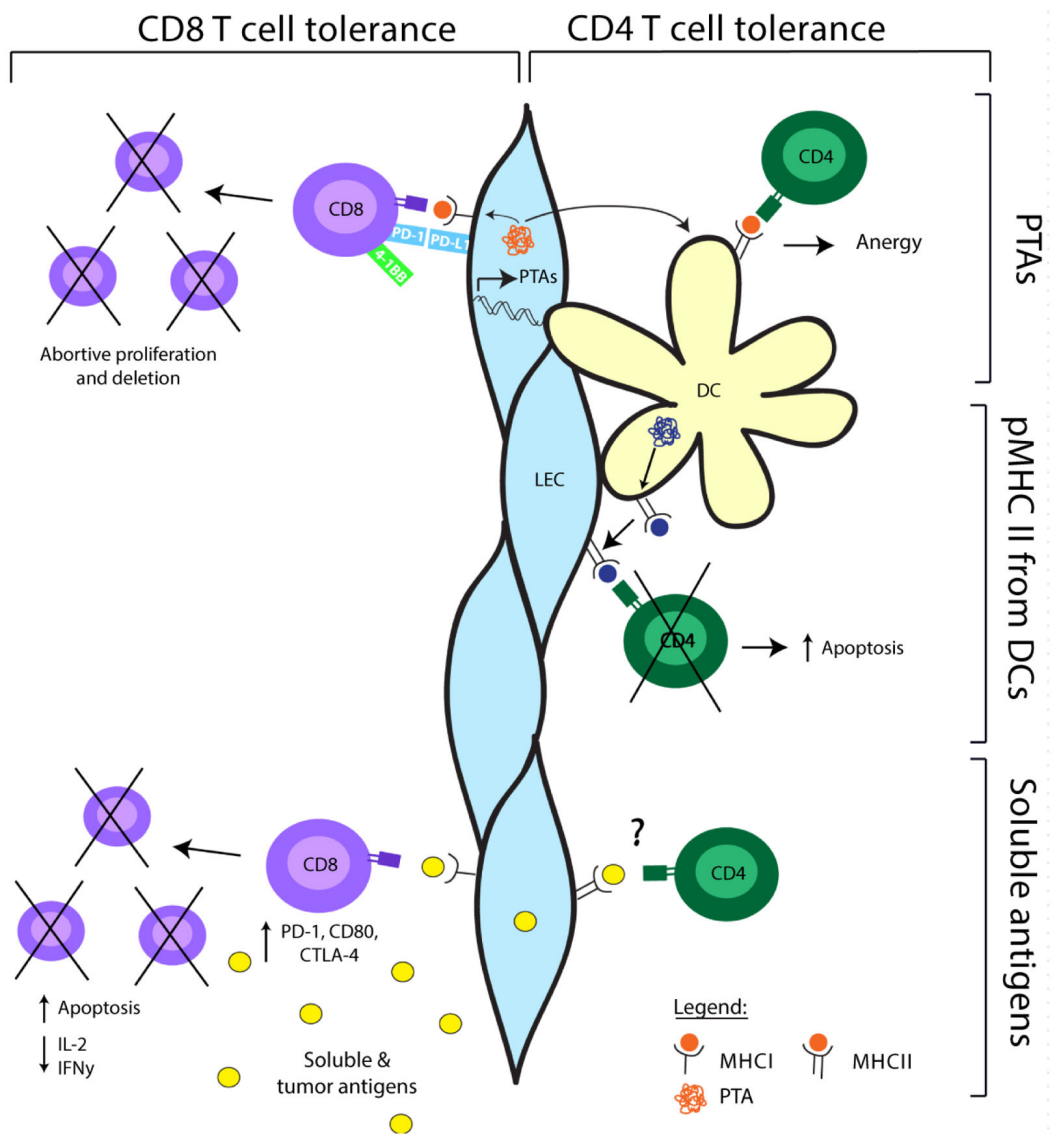
Roles of LEC in CD4 and CD8 T-cell tolerance. LEC transcriptionally express a variety of PTA, which are directly presented to CD8 T-cells, leading to CD8 T-cell proliferation and death through the combined effects of PD-1/PD-L1 signaling and a lack of costimulation. LEC do not directly present PTA on MHC II, but instead transfer the PTA to DC, which induce CD4 T-cell anergy. LEC can acquire peptide/MHC II complexes generated by DC, which are then presented by the LEC, leading to CD4 T-cell apoptosis. Finally, LEC can cross-present soluble antigens from the lymph, leading to dysfunctional CD8 activation and increased apoptosis. It is unknown whether soluble antigens can be presented by LEC on MHC II to CD4 T-cells.

While some of the MHC II expressed on LEC is acquired from DC [49], LEC also express endogenous MHC II [49, unpublished data]. To test whether LEC directly present PTA on their own MHC II molecules and induce CD4 T-cell tolerance, we developed model systems in which the model antigens β-galactosidase (β-gal) and influenza hemagglutinin (HA) were conditionally expressed under the control of the Prox-1 [50] and Lyve-1 [51] promoters (unpublished). An advantage of these models is that both CD8 and CD4 T-cell receptor transgenic mice are available, enabling us to comparatively evaluate the ability of LEC to induce tolerance to epitopes from the same protein presented by either MHC I or MHC II molecules. We found that although LEC directly present β-gal and HA epitopes on MHC I to CD8 T-cells, they do not present either antigen on MHC II to CD4 T-cells *in vivo* or *in vitro*. Our data suggest that the lack of direct presentation of MHC II epitopes by LEC is due to a defect in the MHC II processing and presentation pathway. However, we also found that these antigens were transferred to DC *in vivo*, and subsequent presentation by DC resulted in the induction of CD4 T-cell anergy. Interestingly, this division of labor between LEC and DC is analogous to what occurs in the thymus, where some PTA expressed in mTEC are not directly presented on MHC II, but are instead transferred to DC for the induction of tolerance [52,53]. However, other PTA are directly presented by mTEC on MHC II [52–55]. It is not understood what determines whether or not mTEC will directly present a particular antigen on MHC II. However, mTEC constitutively undergo high levels of autophagy [56,57], suggesting that the efficiency with which antigen can access the autophagy pathway may be a contributing factor [58]. The level of autophagy occurring in LEC at the steady-state has not been examined. Given the ability of LEC to acquire and present tumor-derived and soluble antigens on MHC I [20,21], an important direction for future work is to determine whether these antigens can also be directly loaded onto MHC II in LEC. Regardless, the work performed to date indicates multiple mechanisms by which LECs and DC can cooperate to induce CD4 T-cell tolerance in LN (Figure 1). While DC share pre-formed peptide:MHC II complexes with LEC, the large variety of PTA transcribed by LEC serves as a reservoir and repertoire of antigens that may be acquired by DC for tolerance induction.

## LEC Localized in Peripheral Tissue and LN Subregions Differ in Their Ability to Induce Tolerance

LEC form lymphatic vessels in peripheral tissues in addition to lymphatic sinuses in the LN, raising the question of whether tissue lymphatics share the tolerogenic properties of LEC in the LN (LN-LEC). We found that LEC that form vessels in the diaphragm (D-LEC) and colon (C-LEC) express substantially less tyrosinase mRNA than LN-LEC, and do not induce proliferation of tyrosinase-specific CD8 T-cells *in vitro* [59]. Additionally, 6 out of 7 other PTA tested were more highly expressed in LN-LEC compared to D-LEC or C-LEC. Furthermore, D-LEC and CLEC express substantially less PD-L1 than LN-LEC. Collectively, these results suggest that tolerance induction is a specialized property of LN-LEC not shared by those in tissue lymphatics.

Within the LN, LEC are found in the subcapsular sinus, the cortical sinus, and the medullary sinus. The afferent lymphatics drain into the subcapsular sinus, which forms a thin structure at the outer edge of the LN [60]. DC enter the LN parenchyma through the floor of the subcapsular sinus, while T-cells in the afferent lymph pass through the subcapsular sinus to the medullary sinus, where they enter the LN [61]. To exit the LN, lymphocytes first enter blunt-ended cortical sinuses, which are interspersed throughout the T and B cell zone [62]. Lymphatic fluid in the cortical sinuses flows towards the medullary sinus, and lymphocytes ultimately leave the LN through the medullary sinus in the efferent lymph [62–66]. We showed that LEC in these different sinuses can be distinguished by differential expression of PD-L1, ICAM-1, MAdCAM-1, and LTβR: subcapsular sinus LEC are PD-L1^hi^ICAM-1^hi^MAdCAM-1^+^LTβR^lo^, medullary sinus LEC are PD-L1^hi^ICAM-1^hi^MAdCAM-1^neg^LTβR^+^, and cortical sinus LEC are PD-L1^int^ICAM-1^int^MAdCAM-1^neg^LTβR^+^ [59]. In addition to expressing high levels of PD-L1, medullary LEC are the only subset that expressed a sufficient level of tyrosinase to activate tyrosinase-specific CD8 T-cells. Since the medullary sinus is an exit from the LN, this suggests a model in which LEC function as gatekeepers, engaging and inducing deletion of activated self-reactive CD8 T-cells as they attempt to leave. This model is also intriguing because LEC express the highest level of PD-L1 of any LNSC. A question that remains unaddressed is whether LEC may exert a more generalized quality control role, inducing the deletion of suboptimally activated T-cells, regardless of whether or not the LEC display the cognate antigen for T-cell recognition.

The specific microenvironmental influences that control the phenotypic distinctions between LEC in the periphery and in different LN sinuses remain to be fully understood. Within the LN, we have shown that high level expression of PD-L1 on medullary LEC and MAdCAM-1 on subcapsular sinus LEC is dependent on LTβR signaling and B-cells, but not DC, and that these two signals are independent of one another [59]. Interestingly, the presence of T-cells showed the opposite effect. Tyrosinase expression was not affected by any of these manipulations. We also found that, while a medullary region is present by postnatal day 7, LEC from these neonatal mice do not present tyrosinase and the expression of PD-L1 on LEC is substantially lower than that in adult mice. Combined, these results indicate that the tolerogenic phenotype of LN-LEC develops after the neonatal period in a way that is influenced by, but not entirely dependent on, the effects of lymphocytes and LTβR signaling. Previous work has shown that thymic tolerance is most critical during the perinatal period [67] and that naïve neonatal T-cells directly access peripheral tissues and are tolerized there, instead of in draining LN [68]. This suggests that the relative importance of each tolerogenic site and the associated tolerogenic APC shifts from neonatal to adult animals [69]: during the initial waves of neonatal T-cell development the majority of tolerance occurs in the thymus, and T-cells specific for PTA not expressed in the thymus can be tolerized directly in the peripheral tissues. Later in life, as thymic output decreases and the peripheral tissues become inaccessible to naïve T-cells, peripheral tolerance by LNSC, eTAC, and DC in LN becomes relatively more important to ensure continual tolerance of circulating T-cells.

## Homeostatic Functions of LEC in Immunity

In addition to tolerizing self-reactive T-cells, LEC also influence several other aspects of the immune response. LN and tissue LEC are important sources of IL-7 [70–73], which is an homeostatic survival cytokine for naïve and memory T-cells. IL-7 secretion by LEC is enhanced during lymphopenia, LN remodeling, and revascularization after transplant, and this assists in re-establishing normal LN architecture and cellularity after an infection or other perturbation [71,72]. IL-7 promotes survival and differentiation of memory CD8 T-cells [74,75], suggesting that increased IL-7 production during LN remodeling in the resolution phase of an infection could potentially play a role in enhancing T-cell memory. Additionally, both LN-based and tissue-based LECs express IL-7R and respond to IL-7 in an autocrine fashion, which is required for normal lymphangiogenesis and efficient lymphatic drainage [73]. IL-7R^−/−^ animals have thin and highly branched lymph vessels, suggesting IL-7 might stabilize larger lymphatic vessels. IL-7 also induces VEGF-D secretion in cancer cells, suggesting IL-7 may enhance canonical lymphangiogenesis through the VEGF pathway [76,77]. Through these mechanisms, LEC help control the size of the T-cell compartment.

LEC also control T-cell egress from the LN. Lymphocytes express a receptor for sphingosine-1-phosphate (S1P), a lipid that is present at a relatively high concentration in plasma, but is generally at a low concentration in LN. However, LEC synthesize S1P, providing a high local concentration in their vicinity [51]. Expression of the S1P receptor, S1PR1, on lymphocytes is endocytically downregulated by prolonged exposure to S1P in plasma and by the activation marker CD69 [78,79], but is re-expressed upon entry into LN. Signaling through S1PR1 as lymphocytes encounter LEC allows them to overcome CCR7-mediated retention signals and leave the LN [63,65]. S1P is upregulated when tenascin C, a marker of inflammation induced by a variety of pathogen or damage associated molecular patterns, binds to α9-integrin on LN cortical and medullary sinus LEC, and blockade of α9-integrin inhibits lymphocyte egress [80]. Reciprocal regulation of CD69 and S1P1 prevents activated T-cells from leaving the LN during an immune response, and may also serve a similar function during self-tolerance. Tyrosinase specific T-cells tolerized by LEC undergo several rounds of proliferation before expressing the high-level of PD-1 that mediates deletion. Therefore, retention in the LN may encourage the Tcells to remain in proximity with PD-L1^+^ LEC. Alternatively, self-reactive T-cells may leave their initial activating LN, and encounter PD-L1 on LEC in a downstream LN to complete the tolerogenic process. Regardless, production of IL-7 and S1P are two additional ways that LEC regulate the immune response, by controlling the homeostasis of naïve and memory lymphocytes as well as their ability to exit the LN.

## LEC Restrain T-cell Proliferation in Response to Inflammation

Inflammation has a myriad of effects on lymphatics and the immune response. TLR ligation induces macrophages to secrete VEGF-C/D, which binds to VEGFR-3, triggering proliferation of tissue LEC and lymphangiogenesis [81,82]. IFNγ, TNFα, and TLR ligands increase expression of chemokines and adhesion molecules on LEC, thereby enhancing cell recruitment and migration towards the LN [83,84]. These processes have been extensively reviewed elsewhere [84–87]. Inflammation has the potential to adversely affect tolerance, as inflammatory cytokines such as IFNγ and TNFα can mature DC, leading to the upregulation of costimulatory molecules and the potential for immunogenic presentation of self-antigens acquired in the periphery. LEC help dampen cytokine-induced DC maturation, as TNFα stimulated LEC decrease the expression of the costimulatory molecule CD86 on immature or TNFα stimulated DC and decrease the ability of the DC to stimulate T-cell proliferation [88]. Although the exact mechanism is uncertain, it requires adhesion of the DC to the LEC through ICAM-1/Mac-1 interactions. TNFα stimulated LEC do not affect DC matured with LPS, suggesting that this mechanism only occurs in the absence of pathogen associated molecular patterns, thus potentially contributing to the resolution of inflammation after clearance of infection. LN LEC and FRC also respond to the pro-inflammatory cytokines IFNγ and TNFα by secreting nitric oxide (NO), which limits the proliferation but not effector activity of already activated T-cells [89,90]. Additionally, IFNγ-stimulated cultured human LN-LEC produce indoleamine 2,3 dioxygenase and suppress CD4 T-cell proliferation [91]. Both of these may curtail excessive T-cell expansion to prevent disruption of LN architecture. Interestingly, NO production by FRC also reduced proliferation of self-reactive OT-I CD8 T-cells *in vivo* [90]. Although the effects on deletion were not investigated, this mechanism may also ensure that the proliferating self-reactive T-cells do not expand too rapidly and potentially overwhelm the tolerogenic capacity of the LNSC. However, self-reactive CD8 T-cells generally do not produce IFNγ or TNFα after tolerogenic activation [92] (unpublished data), so the relevance of this mechanism may also vary depending on the characteristics and activation state of the T-cells.

TLR ligation also alters the ability of LEC and FRC to induce tolerance. Primary murine LEC and FRC express TLR3, and treatment with the TLR3 ligand Poly(I:C) upregulates PD-L1 on LEC and FRC but does not change expression of CD80 or CD86 [13]. Poly(I:C) downregulates the PTA OVA in FRC of iFABP-OVA mice, leading to reduced OVA specific CD8 T-cell proliferation *in vitro*; however, the functional consequences *in vivo* were not evaluated. Interestingly, other PTA were either down or up-regulated by TLR3 signaling in both LEC and FRC. PTA downregulation may be an attempt to maintain the ignorance of self-reactive T-cells until after inflammatory conditions have passed, while the upregulation of PD-L1 may help enforce tolerance of any self-reactive T-cells that get activated. The significance of PTA upregulation remains unclear. Additionally, although Poly(I:C) does not upregulate costimulatory molecules on LEC, future work is needed to determine if other inflammatory circumstances can lead to immunogenic activation of T-cells recognizing PTA or endocytosed antigens presented by LEC. Combined, these studies suggest that LN-LEC respond to inflammation by dampening T-cell proliferation, which likely helps ensure continued T-cell tolerance and protects LNSC from damage during an overly vigorous immune response.

## LEC-induced tolerance: a new target for cancer immunotherapy?

Studies performed to date suggest that LEC can enhance tumor growth by either increasing tumor metastasis to the LN through the formation of tumor draining lymphatics [93], or by inducing tolerance of tumor-reactive T-cells [12,20,26,94,95]. This suggests that inhibiting LEC-induced tolerance may provide a method of boosting anti-tumor immunotherapy. Indeed, while tyrosinase is overexpressed in melanoma and is a target of melanoma immunotherapy, LEC-mediated self-tolerance to tyrosinase limits active immunotherapy [95–97]. PD-1 inhibitory antibodies represent one approach to mitigating these effects, and incidentally already show great promise as a monotherapy independently of cancer vaccines in clinical trials [39,98]. These antibodies are currently being tested for their ability to revitalize exhausted effector T-cells and prevent tumor immune evasion [98]. However, our work has established that PD-1 blockade also inhibits LEC-induced tolerance, and this suggests that anti-PD-1 blockade may particularly complement efforts to specifically target tyrosinase using cancer vaccines or T-cell adoptive therapy. This combination therapy may provide a synergistic benefit by inhibiting tolerance and simultaneously preventing T-cell exhaustion. Increasing our understanding of the role of LEC in T-cell tolerance may provide new opportunities to enhance cancer immunotherapies.

## Technical Challenges and Opportunities in LEC Research

Studying the role of LEC in tolerance and immunity presents a number of challenges due to the rarity and fragility of this cell type. LEC represent about 0.2% of the cells in the LN, and on average less than 50,000 LEC are isolated from the major LN per mouse [13,59]. The small numbers of LECs makes many biochemical techniques impractical on *ex vivo* samples. As a result, several groups have optimized protocols to expand LNSC and LEC in culture [99,100]. However, the phenotype of LEC changes rapidly in culture, as the surrounding microenvironment contributes to the maintenance of LEC differentiation *in vivo* [59,101–103]. Notably, we have found that mRNA levels of the PTA tyrosinase and Mart1 diminish more than 50-fold, and expression of PD-L1 and HVEM is lost after 5 days in culture (unpublished). Additionally, the geography of the LN and the localization of LEC at the LN entrances and exits may affect how LEC interact with other cells, and the physiological roles of LEC in the LN. This information is lost *in vitro*. As a result, it is important to confirm the physiological relevance of all *in vitro* observations with an *in vivo* system. Fortunately, a number of mouse models have recently become available to facilitate *in vivo* research. LEC are a radioresistant cell, so bone marrow chimeras can be used to localize genetic knockouts to either the hematopoietic or radioresistant compartments. Several groups have also developed mice expressing Cre-recombinase in LEC, under either the control of the Lyve-1 [51], Prox1 [50,104], or podoplanin [105] promoters. These mice can be used to specifically delete floxed genes in LEC. Additionally, reporter mice expressing tdTomato [106], mOrange2 [107], or GFP [108] under the control of the Prox1 promoter allows for intravital two-photon imaging and lymphatic tracing. These tools will provide new opportunities to identify, track, and manipulate LEC *in vivo*.
